# Clinical and immunological evaluation of anti-apoptosis protein, survivin-derived peptide vaccine in phase I clinical study for patients with advanced or recurrent breast cancer

**DOI:** 10.1186/1479-5876-6-24

**Published:** 2008-05-10

**Authors:** Tetsuhiro Tsuruma, Yuji Iwayama, Tosei Ohmura, Tadashi Katsuramaki, Fumitake Hata, Tomohisa Furuhata, Koji Yamaguchi, Yasutoshi Kimura, Toshihiko Torigoe, Nobuhiko Toyota, Atsuhito Yagihashi, Yoshihiko Hirohashi, Hiroko Asanuma, Kumiko Shimozawa, Minoru Okazaki, Yasuhiro Mizushima, Naohiro Nomura, Noriyuki Sato, Koichi Hirata

**Affiliations:** 1 Dept. of Surgery, Sapporo Medical University School of Medicine, Sapporo, Japan; 2 Dept. of Pathology, Sapporo Medical University School of Medicine, Sapporo, Japan; 3 Dept. of Laboratory Diagnosis, Sapporo Medical University School of Medicine, Sapporo, Japan; 4 Japan Science and Technology Corporation Innovation Plaza Hokkaido, Sapporo, Japan; 5 Dept. of Surgery, Sapporo Nyusen Geka Clinic, Sapporo, Japan; 6 Dept. of Surgery, Ashibetsu Municipal Hospital, Ashibetsu, Japan; 7 Dept. of Medicine, Kitahiroshima Hospital, Kitahiroshima, Japan

## Abstract

**Background:**

We previously reported that survivin-2B, a splicing variant of survivin, was expressed in various types of tumors and that survivin-2B peptide might serve as a potent immunogenic cancer vaccine. The objective of this study was to examine the toxicity of and to **c**linically and immunologically evaluate survivin-2B peptide in a phase I clinical study for patients with advanced or recurrent breast cancer.

**Methods:**

We set up two protocols. In the first protocol, 10 patients were vaccinated with escalating doses (0.1–1.0 mg) of survivin-2B peptide alone 4 times every 2 weeks. In the second protocol, 4 patients were vaccinated with the peptide at a dose of 1.0 mg mixed with IFA 4 times every 2 weeks.

**Results:**

In the first protocol, no adverse events were observed during or after vaccination. In the second protocol, two patients had induration at the injection site. One patient had general malaise (grade 1), and another had general malaise (grade 1) and fever (grade 1). Peptide vaccination was well tolerated in all patients. In the first protocol, tumor marker levels increased in 8 patients, slightly decreased in 1 patient and were within the normal range during this clinical trial in 1 patient. With regard to tumor size, two patients were considered to have stable disease (SD). Immunologically, in 3 of the 10 patients (30%), an increase of the peptide-specific CTL frequency was detected. In the second protocol, an increase of the peptide-specific CTL frequency was detected in all 4 patients (100%), although there were no significant beneficial clinical responses. ELISPOT assay showed peptide-specific IFN-γ responses in 2 patients in whom the peptide-specific CTL frequency in tetramer staining also was increased in both protocols.

**Conclusion:**

This phase I clinical study revealed that survivin-2B peptide vaccination was well tolerated. The vaccination with survivin-2B peptide mixed with IFA increased the frequency of peptide-specific CTL more effectively than vaccination with the peptide alone, although neither vaccination could induce efficient clinical responses. Considering the above, the addition of another effectual adjuvant such as a cytokine, heat shock protein, etc. to the vaccination with survivin-2B peptide mixed with IFA might induce improved immunological and clinical responses.

## Background

The incidence of breast cancer has continuously increased in Japan, similar to European countries and the USA, whereas mortality from breast cancer has declined, indicating improving survival because of the development of early diagnosis [1-3]. However, metastatic recurrence still occurs, and once the cancer has spread beyond the breast and locoregional nodal areas it is felt to be incurable [4]. In the case of metastatic recurrence, the prevailing treatment is systemic chemotherapy, which is fraught with various adverse effects. Thus, we considered the availability of immunotherapy, which is generally reported to be safe, for advanced or recurrent breast cancer.

Tumor cells express antigens that can be recognized by the host's immune system. In the past decade, many antigenic peptides, which can be recognized by CTLs, have been identified [5-9]. As a result, clinical trials of peptide-based immunotherapy for cancer have taken place. Melanoma antigen peptides were the first to be tested in phase I and phase II studies for active immunization of metastatic melanoma patients [10,11]. Recently, there are reports of clinical trials for various cancers, including colorectal cancer [12], esophageal cancer [13], pancreatic cancer [14], among others. However, most clinical trials did not demonstrate sufficient anti-tumor clinical responses. Thus, it is necessary to establish peptide-based immunotherapy that can induce sufficient clinical responses.

Survivin was initially isolated as one of the inhibitors of the apoptosis protein family with only one baculovirus inhibitor of apoptosis protein (IAP) repeat domain [15]. Survivin is aberrantly expressed in various cancer cells but is undetectable in normal differentiated adult tissues, with the exception of the testis, thymus and placenta. We have previously reported that survivin-2B, a splicing variant of survivin, is expressed in various tumor cell lines [16], and the survivin-2B80-88 (AYACNTSTL) peptide derived from the exon 2B-encoded region is recognized by CD8+ CTLs in the context of HLA-A24 molecules [16]. In addition, we recently reported further evidence that survivin-2B80-88 peptide might serve as a potent immunogenic cancer vaccine for various cancer patients [17]. In that report, we demonstrated that overexpression of survivin was detected in surgically resected primary tumor specimens of most breast cancers in an immunohistochemical study. In addition, HLA-A24/survivin-2B80-88 tetramer analysis revealed that there were an increased number of CTL precursors in peripheral blood mononuclear cells (PBMCs), and in vitro stimulation of PBMCs from 6 breast cancer patients with survivin-2B80-88 peptide led to increases of the CTL precursor frequency. Furthermore, CTLs specific for this peptide were successfully induced in PBMCs from all 7 HLA-A24^+ ^patients (100%) with breast cancers and exhibited cytotoxicity against HLA-A24^+^/survivin^+ ^adenocarcinoma cells [17]. On the basis of these studies, we started a phase I clinical study of vaccination with survivin-2B peptide for patients with advanced or recurrent breast cancer.

## Methods

### Patient selection

The study protocol was approved by the Clinic Institutional Ethical Review Board of the Medical Institute of Bioregulation, Sapporo Medical University, Japan. All patients gave informed consent before being enrolled. Patients enrolled in this study were required to conform to the following criteria: (1) to have histologically confirmed breast cancer, (2) to be HLA-A*2402 positive, (3) to be survivin-positive in the carcinomatous lesions by immunohistochemistry, (4) to be between 20 and 85 years old, (5) to be unresectable advanced cancer or recurrent cancer and (6) to have Eastern Cooperative Oncology Group (ECOG) performance status between 0 and 3. Exclusion criteria included (1) prior cancer therapy such as chemotherapy, radiation therapy, steroid therapy, or other immunotherapy within the past 4 weeks, (2) the presence of other cancers that might influence the prognosis, (3) immunodeficiency or a history of splenectomy, (4) severe cardiac insufficiency, acute infection, or hematopoietic failure, (5) ongoing breast-feeding and (6) unsuitability for the trial based on clinical judgment. This study was carried out at the Department of Surgery, Sapporo Medical University Primary Hospital from July 2003 through November 2005.

### Peptide preparation

The peptide, survivin-2B80-88 with the sequence **AYACNTSTL**, was prepared under good manufacturing practice conditions by Multiple Peptide Systems (San Diego, CA). The identity of the peptide was confirmed by mass spectrometry analysis, and the purity was shown to be more than 98% as assessed by high pressure liquid chromatography analysis.

The peptide was supplied as a freeze-dried, sterile white powder. It was dissolved in 1.0 ml of physiological saline (Otsuka Pharmaceutical Co., Ltd., Tokyo, Japan) and stored at -80°C until just before use.

### Incomplete Freund's Adjuvant (IFA) preparation

Montanide ISA 51 (SEPPIC Inc., NJ, USA) was used as incomplete Freund's adjuvant (IFA).

### Patient treatment

In this phase I clinical study, two protocols were used. One was a basic protocol, namely, with survivin-2B peptide alone, and the other used survivin-2B peptide mixed with IFA. In this trial, the primary endpoint was safety. The second endpoint was investigations about anti-tumor effects and clinical and immunological monitoring.

In the first protocol, the vaccination schedule was as follows. Vaccinations with survivin-2B peptide were given subcutaneously (s.c.) four times at 14-day intervals. To set up a dose-escalation trial, the patients were separated into the following two groups: in group 1 patients were vaccinated with 0.1 mg of the peptide and in group 2 patients were vaccinated with 1.0 mg of the peptide. Each group included five patients. Escalation to the next dose was allowed if side effects did not exceed grade 3. If patients whose disease was not far advanced hoped for continuation of this peptide vaccination therapy, we vaccinated them in the same manner after the fourth vaccination.

In the second protocol, survivin-2B peptide at a dose of 1 mg/1 ml and IFA at a dose of 1 ml were mixed immediately before vaccination. Then the patients were vaccinated subcutaneously (s.c.) four times at 14-day intervals.

### Delayed-type hypersensitivity (DTH) skin test

A DTH skin test was performed at each vaccination. The peptide (10 μg) solution in physiological saline (0.1 ml) or physiological saline alone (0.1 ml) was separately injected intradermally (i.d.) into the forearm opposite the vaccination site. A positive reaction was defined as a more than 4 mm diameter area of erythema and induration 48 hr after the injection.

### Toxicity evaluation

Patients were examined closely for signs of toxicity during and after vaccination. Adverse events were recorded using the National Cancer Institute Common Toxicity Criteria (NCI-CTC).

### Clinical response evaluation

Physical examinations and hematological examinations were conducted before and after each vaccination. Tumor markers (CEA, CA15-3, NCC-ST-439, and ICTP) were examined monthly.

Tumor size was evaluated by computed tomography (CT) scans or MRI in comparison with the size before the first vaccination and that after the fourth vaccination. A complete response (CR) was defined as complete disappearance of all measurable and evaluable disease. A partial response (PR) was defined as a ≧30% decrease from the baseline in the size of all measurable lesions (sum of maximal diameters). Progressive disease (PD) was defined as an increase in the sum of maximal diameters by at least 20% or the appearance of new lesions. Stable disease (SD) was defined as the absence of criteria matching those for CR, PR, or PD. Patients who received less than four vaccinations were excluded from all evaluations in this study.

### *In vitro *stimulation of PBMC

PBMCs were isolated from blood samples by Ficoll-Conray density gradient centrifugation. Then they were frozen and stored at -80°C. As needed, frozen PBMCs were thawed and incubated in the presence of 30 μl/ml survivin-2B peptide in AIM-V medium containing 10% human serum at room temperature. Next, interleukin-2 was added at a final concentration of 50 U/ml 1 hr, 2 days, 4 days and 6 days after the addition of the peptide. On day 7 of culture, the PBMCs were analyzed by tetramer staining and ELISPOT assay.

### Tetramer staining

FITC-labeled HLA-A*2402-HIV peptide (RYLRDQQLL) tetramer and PE-labeled HLA-A*2402-Survivin-2B80-88 peptide tetramer were purchased from MBL Inc.(Japan). For flow cytometric analysis, PBMCs, which were stimulated *in vitro *as above, were stained with the tetramers at 37°C for 20 min, followed by staining with FITC- or PerCP-conjugated anti-CD8 mAb (Beckton Dickinson Biosciences) at 4°C for 30 min. Cells were washed twice with PBS before fixation in 1% formaldehyde. Flow cytometric analysis was performed using FACSCalibur and CellQuest software (BD Biosciences). The frequency of CTL precursors was calculated as the number of tetramer-positive cells divided by the number of CD8-positive cells.

### ELISPOT assay

ELISPOT plates were coated sterilely overnight with an IFN-γ capture antibody (Beckton Dickinson Biosciences) at 4°C. The plates were then washed once and blocked with AIM-V medium containing 10% human serum for 2 hr at room temperature. CD8-positive T cells separated from patients' PBMCs (5 × 10^3 ^cells/well), which were stimulated *in vitro *as above, were then added to each well along with HLA-A24-transfected CIR cells (CIR-A24) (5 × 10^4 ^cells/well), which had been preincubated with or without survivin-2B80-88 peptide (10 μg/ml) and with an HIV peptide as a negative control. After incubation in a 5% CO_2 _humidified chamber at 37°C for 24 hours, the wells were washed vigorously five times with PBS and incubated with a biotinylated anti-human IFN-γ antibody and horseradish peroxidase-conjugated avidin. Spots were visualized and analyzed using KS ELISPOT (Carl Zeiss, Germany).

## Results

### Patient profiles

In the first protocol with survivin-2B peptide alone, 12 patients were initially enrolled in the study (Additional file 1), but two (cases 7 and 10) discontinued halfway through the protocol. One patient (case 7) had local recurrence, brain and lung metastases from bilateral breast cancer and was removed from the study after 3 vaccinations since new brain metastasis appeared and she required radiation therapy. Another patient (case 10) had lymph node metastases from right breast cancer. She was removed from the study after 3 vaccinations because of enlargement of lymph node metastases. Neither of the treatment interruptions was due to adverse effects of the vaccination. Ten patients received the complete regimen including four vaccinations and were evaluated. They were all women, whose average age was 49 years (range, 34–71).

In the second protocol with survivin-2B peptide mixed with IFA, five patients were initially enrolled in the study (Additional File 2), but one (case 2) discontinued halfway through the protocol. This patient had lung and liver metastases from right breast cancer and was removed from the study after 3 vaccinations because of exacerbated liver function resulting from advanced liver metastases. In this protocol, there were no patients who dropped out because of adverse events due to the peptide vaccination. Four patients received the complete regimen including four vaccinations and were evaluated. They were all women, whose average age was 52 years (range, 36–71).

### Safety

Peptide vaccination was well tolerated in all patients. In patients vaccinated with the peptide alone, no adverse events were observed during or after vaccination (Additional File 3). Of the patients vaccinated with the peptide mixed with IFA, two (cases 1 and 3) had induration at the injection site (Additional File 4). One (case 4) had general malaise (grade 1) and one (case 5) had general malaise (grade 1) and fever (grade 1). No other severe adverse events were observed during or after vaccination.

### Clinical responses

**Figure 1:**
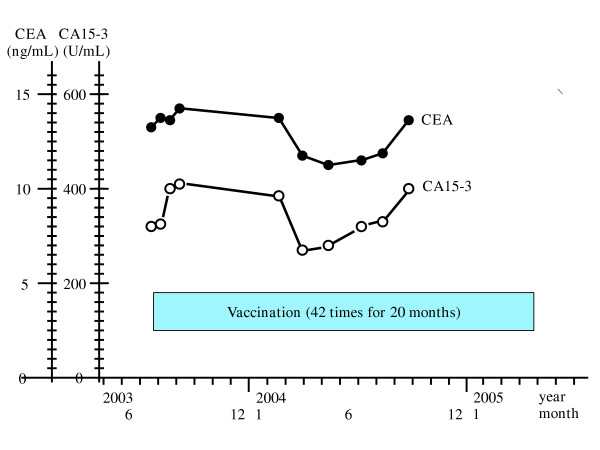
**The changes in tumor marker levels in case 3 in the first protocol**. For case 3, with bone and lymph node metastases, vaccination continued 42 times for 20 months. In this period new metastatic foci did not appear and there was almost no increase in size of the metastatic lesions. Tumor marker levels did not increase rapidly.

Table 3 summarizes the clinical outcomes for the 10 patients in the first protocol with survivin-2B peptide alone. In all patients except two (cases 1 and 9), the tumor marker levels were increased. In one patient (case 1), the levels of CEA, CA15-3 and NCC-ST-439 were within the normal range during this clinical trial. The level of ICTP was decreased from 7.2 ng/ml just before the first vaccination to 5.5 ng/ml after the fourth vaccination. However, this change was not considered a significant decrease. In case 9, all tumor marker levels were within the normal range during this clinical trial. As for tumor size, two patients (cases 3 and 5) were considered to have SD. In one patient (case 9) who had bone metastases, the area of bone metastases did not increase in bone scintigraphy, and new metastatic foci did not appear during this trial. In this patient, although there was no aggravation of the clinical condition, we could not estimate the clinical response by our criteria because bone metastases were not able to be evaluated in CT images. The other patients were considered to have progressive disease (PD). In this protocol, if patients whose disease was not far advanced hoped for the continuation of this peptide vaccination therapy, we vaccinated them in the same manner after the fourth vaccination. There were 2 patients (cases 3 and 9) who were vaccinated for more than one year. In case 3, with bone and lymph node metastases, vaccination continued 42 times for 20 months (Fig. 1). For this period, new metastatic foci did not appear and there was almost no increase in the size of the metastatic lesions in this patient. Tumor marker levels did not increase rapidly (Fig. 1). In addition, she maintained good quality of life because there was no adverse effect.

Table 4 summarizes the clinical outcomes for the 4 patients in the second protocol with survivin-2B peptide mixed with IFA. In all patients, the tumor marker levels were increased. As for tumor size, all patients were considered to have PD.

### DTH skin test

A DTH skin test was performed at each vaccination and assessed 48 hr later. A positive reaction was defined as erythema and induration more than 4 mm in diameter. In the first protocol with survivin-2B peptide alone, 2 of the 10 patients (20%) exhibited a positive DTH reaction at least once during the study. In the second protocol with peptide mixed with IFA, a positive DTH reaction was observed in 1 of the 4 patients (25%).

### Tetramer staining assay and ELISPOT assay

**Figure 2:**
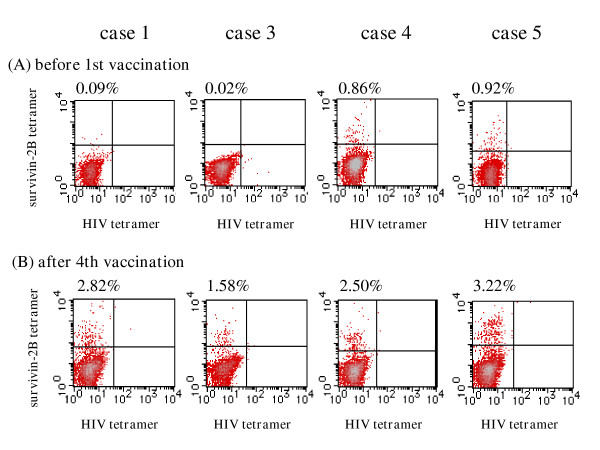
**Tetramer staining before the first vaccination and after the fourth vaccination in the second protocol**. FITC-labeled HLA-A*2402-HIV peptide (RYLRDQQLL) tetramer and PE-labeled HLA-A*2402-Survivin-2B80-88 peptide tetramer were uesd. For flow cytometric analysis, PBMCs, which were stimulated *in vitro*, were stained with the tetramers at 37°C for 20 min, followed by staining with FITC- or PerCP-conjugated anti-CD8 mAb (Beckton Dickinson Biosciences) at 4°C for 30 min. Cells were washed twice with PBS before fixation in 1% formaldehyde. Flow cytometric analysis was performed using FACSCalibur and CellQuest software (BD Biosciences). The frequency of CTL precursors was calculated as the number of tetramer-positive cells divided by the number of CD8-positive cells. The peptide-specific CTL frequency is indicated as the percentage of tetramer-positive CTL cells among CD8-positive T cells before the first vaccination and after the fourth vaccination. The peptide-specific CTL frequency after the fourth vaccination (B) was compared with that before the first vaccination (A). In the second protocol with the peptide mixed with IFA, the peptide-specific CTL frequency was increased in all 4 patients (100%).

To determine if the survivin-2B peptide vaccination could bring about specific immune responses in the patients, we analyzed the peptide-specific CTL frequency by using the HLA-A24/peptide tetramer. The change of tetramer-positive CTL frequency was evaluated by comparison with that before the first vaccination and that after the fourth vaccination as follows: detected and undetected. Detected was defined as an increase of twofold or more. Undetected was defined as a less than twofold increase. In the first protocol with the peptide alone, a change was considered to be detected in 3 patients (30%) (Table 3). On the other hand, in the second protocol with peptide mixed with IFA, it was considered to be detected in all 4 patients (100%) (Table 4). In Figure 2, the peptide-specific CTL frequency in the second protocol is indicated as the percentage of tetramer-positive CTL cells among CD8-positive T cells before the first vaccination and after the fourth vaccination.

**Figure 3:**
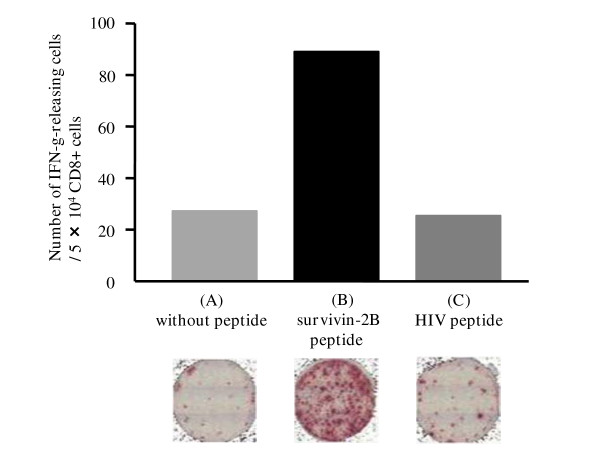
**ELISPOT assay after the fourth vaccination of case 5 in the second protocol**. In the wells that were preincubated without survivin-2B peptide (A) or with an HIV peptide (C), spots were almost not visualized. On the other hand, in the wells that were preincubated with survivin-2B peptide (B), many spots were visualized. These findings demonstrate that CD8-positive T cells separated from the patients' PBMCs had a peptide-specific IFN-γ response.

ELISPOT assay of CD8-positive T cells separated from the patients' PBMCs showed peptide-specific IFN-γ responses in 2 patients, one of whom (case 8) was in the first protocol while the other (case 5) was in the second protocol. To be more precise, in case 5 in the second protocol, in the wells that were preincubated without the survivin-2B peptide and with the HIV peptide, spots were almost not visualized. On the other hand, in the wells that had been preincubated with survivin-2B peptide, many spots were visualized (Fig. 3). In these two patients, the peptide-specific CTL frequency was also increased by the vaccination with survivin-2B peptide.

## Discussion

Recently, a large number of tumor antigens and epitopes recognized by CTLs have been identified, and reports of clinical trials utilizing peptide vaccination are increasing [10,18-20]. We demonstrated that survivin was expressed in a large proportion of various cancer specimens, and the survivin-2B-derived peptide could induce a CTL response in the context of HLA-A24 [16,17]. In addition, we showed an elevation in CTL precursor frequencies in PBMCs of HLA-A24+ cancer patients by using an HLA-A24/survivin-2B peptide tetramer. On the basis of the above studies, we started a phase I clinical study of survivin-2B peptide vaccine therapy for patients with advanced or recurrent colorectal cancer in 2003 [12]. In this study, we vaccinated patients with survivin-2B peptide alone, and reported the safety of this peptide vaccination and the potency of anti-tumor effects induced by the peptide vaccination. At that time, we started this phase I clinical study of peptide vaccine therapy for patients with advanced or recurrent breast cancer as well. In this study, we vaccinated patients with not only survivin-2B peptide alone but also survivin-2B mixed with IFA in order to induce greater anti-tumor effects. We recently established immunological monitoring methods using a tetramer staining assay and ELISPOT assay. Thus, in this study, we also conducted immunological monitoring using these techniques.

Survivin is an ideal tumor-associated antigen expressed in a broad variety of malignancies and recognized by specific cytotoxic T cells [21]. The first survivin-derived peptides were characterized in 2000 [22,23]. Subsequently, there have been many reports about survivin peptide responses. Grube et al. [24] reported that HLA-A2.1-restricted survivin peptide induced CD8+ T cell reactivity in patients with multiple myeloma. Andersen et al. [25] reported the detection of HLA-A24 restricted and survivin peptide-specific CD8-positive cells by IFN-γ ELISPOT assay and perforin ELISPOT assay in patients with breast cancer, melanoma and renal cancer. Abrogating the function of survivin not only limits the proliferative potential and viability of tumor cells directly [26], but also inhibits tumor angiogenesis [27]. Xiang et al. [28] reported that a DNA vaccine targeting survivin lead to eradication of pulmonary metastases by a combinational effect inducing tumor cell apoptosis and suppressing tumor angiogenesis in a murine model. Thus, survivin is a suitable target for immune therapy for cancer [26,29]. Recently, a number of survivin epitopes restricted to several additional HLA-molecules have been identified [22,25,29-34], and several clinical trials of immunotherapy based on survivin-derived peptides have been initiated. Wobser et al. [35] reported complete remission of liver metastasis of pancreatic cancer under vaccination with an HLA-A2 restricted survivin peptide. In addition, a phase I/II trial with HLA-A1, -A2 and -B35 restricted survivin peptides for patients with advanced cancer is ongoing. Fuessel et al. [27] reported a phase I clinical trial for patients with prostate cancer in which they evaluated a vaccination with DCs loaded with five different prostate cancer-associated antigens (survivin, prostate-specific antigen [PSA], prostate-specific membrane antigen [PSMA], and prostein, transient receptor potential p8 [trp-p8]) and concluded that the concept was safe and feasible. Besides the above-mentioned investigations, various clinical trials are ongoing now.

At present, 4 splicing variants of survivin (survivin-ΔEx3, survivin-2α, survivin-2B, and survivin-3B) have been identified. Espinosa et al. [36] reported that the expression of survivin-ΔEx3 and survivin-2B was higher in cervical cancer samples. There is also a report that survivin-2B was dominantly expressed in gastric cancer [37]. Futakuchi et al. [38] reported that the ratios of survivin-2B/survivin and survivin-ΔEx3/survivin in malignant cervical tissue samples were significantly higher than those in normal cervical tissue templates. Moreover, the ratio of survivin-2B/survivin was increased in patients with higher stages and with pelvic lymph node metastasis. These reports might support the idea that survivin-2B is the ideal target of immunotherapy for cancer patients [17,39], especially for those with advanced or recurrent cancer. On the other hand, Mahotka et al. [40] reported that the ratio of survivin-2B/survivin was decreased in the late stages of renal cell carcinoma. Yamada et al. [41] reported that there was no significant difference in the ratio of survivin-2B/survivin in malignant brain tumors and gliomas compared with nonglioma. There is a hypothesis that the relevant ratios of the survivin and splicing variants may regulate the ultimate apoptotic activities of cancer cells and determine their biological behaviours and responses to apoptosis-inducing treatment [37,40]. Nevertheless, the exact roles and expression of survivin splicing variants and their interplay in various cancers are as yet unclear because of the high complexity of its regulation [36,42]. We previously demonstrated that the expression of survivin-2B was detected in a variety of tumor cell lines but not in normal tissues except in the thymus, although low levels of survivin expression were detected by reverse transcription-PCR analysis [16]. In addition, we reported that survivin-2B-specific CTLs could be induced efficiently from PBMCs of HLA-A24-positive survivin-positive cancer patients [17]. As described above, we are sure that survivin-targeting immunotherapy with survivin-2B peptide should be a reasonable strategy.

A dose-escalation trial was chosen to estimate a safe and optimal dose in the first protocol with survivin-2B peptide alone. We used 0.1 mg and 1.0 mg dosage groups, each consisting of five patients. No adverse events were observed in either group. In addition, for the patients (cases 3 and case 9) who were vaccinated 42 times and 38 times respectively, adverse events were not observed during or after the vaccination. Thus, we concluded that the survivin-2B peptide vaccine was safe and could be repeatedly injected into patients without severe adverse events. In comparison between patients who were vaccinated with 0.1 mg and 1.0 mg of the peptide, there was almost no difference in clinical responses. However, peptide-specific immune responses in tetramer staining and ELISPOT assay were frequently induced in patients vaccinated with 1.0 mg of the peptide in comparison with patients vaccinated with 0.1 mg of the peptide. Therefore, we decided that the optimal dose of the peptide was 1.0 mg. IFA has sustained-release effect, which can enhance the anti-tumor effect of the peptide injected subcutaneously. So, in the second protocol which its purpose was to induce the more effective anti-tumor effect by the survivin-2B peptide, IFA was used mixed with 1.0 mg of the peptide. In this protocol, two patients (cases 1 and 3) had induration. This was due to IFA trapped in the subcutaneous lesion. Patient 4 had general malaise (grade 1), and patient 5 had general malaise (grade 1) and fever (grade 1). No other severe adverse events were observed during or after vaccination. Therefore, we concluded that the vaccine using survivin-2B peptide mixed with IFA was safe, as was the peptide alone.

Positive delayed-type hypersensitivity (DTH) reactions were observed in 2 of the 10 patients (20.0%) in the first protocol and in 1 of the 4 patients (25.0%) in the second protocol at least once during the vaccination. Some reports have suggested a positive correlation between DTH and clinical [43] or immunological responses [44]. In this study, in case 5 in the first protocol a positive DTH reaction was observed and the change of tumor size was considered to indicate SD, while the tumor marker level was considered to have increased, although immunological responses were not induced. However, neither clinical nor immunological responses were totally associated with a positive DTH reaction in this study.

In the first protocol with survivin-2B peptide alone, none of patients in the 0.1 mg peptide group had tetramer response and that 3 of the 5 patients (30%) in the 1.0 mg peptide group had increased the tetramer-specific CTL frequency. On the other hand, in the second protocol with survivin-2B peptide mixed with IFA, all patients had a significant increase of the tetramer-positive CTL frequency. These results might demonstrate that the addition of IFA could enhance the immunological responses to the survivin-2B peptide. In addition, these findings might also indicate that the addition of another effectual adjuvant such as a cytokine, heat shock protein [45], etc. to the vaccination with survivin-2B peptide mixed with IFA could more effectively enhance the immunological and clinical responses to the peptide. At present a phaseII clinical study of survivin-2B peptide vaccine therapy, in which the peptide is combined with IFA and IFN-α, is ongoing in our group.

In the second protocol with the peptide mixed with IFA, although all patients had an increase of the tetramer-positive CTL frequency, only one patient had a peptide specific IFN-γ response in the ELISPOT assay. In tetramer staining, the frequency of the peptide-specific CTL was investigated. In the ELISPOT assay, the function of the peptide-specific CTL was investigated. It is possible that the peptide-specific CTL induced by the vaccination might not function well due to immune escape mechanisms in the effector phase. Thus, this might be one of the reasons why the CTL response to the vaccination was not sufficient to induce clinical responses. This also could imply a dysfunction of the host immune system or an immunosuppressive effect of the tumor microenvironment, including the down-regulation of HLA-class I molecules on tumor cells. Therefore, we have recently begun to investigate a novel strategy to overcome the immune escape in peptide vaccine therapy.

## Conclusion

In conclusion, this phase I clinical study revealed that the administration of not only survivin-2B peptide alone but also the peptide in combination with IFA for patients with advanced or recurrent breast cancer was well tolerated. Vaccination with survivin-2B peptide mixed with IFA increased the frequency of the peptide-specific CTL more effectively than vaccination with the peptide alone, although neither vaccination could induce an efficient clinical response. Thus, the addition of IFA might enhance the immunological response to the peptide vaccination. Considering the above, the addition of another effectual adjuvant such as a cytokine, heat shock protein [45], etc. to the vaccine using survivin-2B peptide mixed with IFA might induce improved immunological and clinical responses.

## List of abbreviations

PBMCs: peripheral blood mononuclear cells, CTL: cytotoxic T lymphocyte, IAP: inhibitor of the apoptosis proteins, ECOG: Eastern Co-operative Oncology Group, IFA: incomplete Freund's adjuvant, DTH: delayed-type hypersensitivity, CR: complete response, PR: partial response, SD: stable disease, PD: progressive disease.

## Competing interests

The authors declare that they have no competing interests.

## Authors' contributions

TT^1^ performed peptide vaccine preparation, and contributed largely to designing the phase I clinical study, coordination of this study and analysis of all results. TT^1^ and YI contributed to the medical examination and vaccination of patients, and interpretation of clinical data. TO, TK, FH, TF, KY, YK and NT contributed to the medical care. TT2 and YH contributed to designing the peptide vaccine. TT^2^ contributed to interpretation of immunological data. TT^2^, AY, HA and KS contributed to analysis of immunological responses, such as tetramer staining, ELISPOT assay, etc. MO, YM, NN contributed to registration of patients. NS and KH contributed largely to control over the clinical system and immunological study, respectively, and they also contributed to reviewing the manuscript. All authors have read and approved the final manuscript. (TT^1^: Tetsuhiro Tsuruma,  TT^2^: Toshihiko Torigoe)

## Supplementary Material

Additional file 1 Table 1: Profiles of patients enrolled in the first protocol with survivin-2B peptide alone. The data showed profiles of patients enrolled in the first protocol with survivin-2B peptide alone. Click here for file

Additional file 2 Table 2: Profiles of patients enrolled in the second protocol with survivin-2B peptide mixed IFA. The data showed profiles of patients enrolled in the second protocol with survivin-2B peptide mixed IFA. Click here for file

Additional file 3 Table 3: Outcome in the first protocol with survivin-2B peptide alone. This data showed the clinical and immunological evaluation in the first protocol. Click here for file

Additional file 4 Table 4: Outcome in the second protocol with survivin-2B peptide mixed IFA. This data showed the clinical and immunological evaluation in the second protocol. Click here for file
