# Supplementary material for: Clinical and immunological evaluation of anti-apoptosis protein, survivin-derived peptide vaccine in phase I clinical study for patients with advanced or recurrent breast cancer
Source: J Transl Med. 2008 May 10;6:24. doi: 10.1186/1479-5876-6-24 (PMC2430193; doi:10.1186/1479-5876-6-24)
Supplement: Additional file 1 — Table 1: Profiles of patients enrolled in the first protocol with survivin-2B peptide alone. The data showed profiles of patients enrolled in the first protocol with survivin-2B peptide alone. [file 1479-5876-6-24-S1.pdf]

Table 1 : Profiles of patients enrolled in the first protocol with survivin-2B peptide alone

patient no.	age	sex	site of metastases
1	60	F	brain
2	42	F	lymph node, lung
3	50	F	lymph node, bone
4	34	F	lymph node, lung, liver, bone
5	71	F	local, lung
6	40	F	lymph node
7	70	F	local, brain, lung
8	46	F	lymph node, brain, lung
9	58	F	bone
10	51	F	lymph node
11	51	F	lymph node, lung, liver, bone
12	38	F	local, lymph node, brain, lung, bone
