# Supplementary material for: Clinical and immunological evaluation of anti-apoptosis protein, survivin-derived peptide vaccine in phase I clinical study for patients with advanced or recurrent breast cancer
Source: J Transl Med. 2008 May 10;6:24. doi: 10.1186/1479-5876-6-24 (PMC2430193; doi:10.1186/1479-5876-6-24)
Supplement: Additional file 2 — Table 2: Profiles of patients enrolled in the second protocol with survivin-2B peptide mixed IFA. The data showed profiles of patients enrolled in the second protocol with survivin-2B peptide mixed IFA. [file 1479-5876-6-24-S2.pdf]

Table 2 :
Profiles of patients enrolled in the second protocol with survivin-2B peptide mixed IFA

patient no.	age	sex	site of metastases
1	71	F	lymph node, lung, liver, bone
2	52	F	lung, liver
3	36	F	lymph node
4	53	F	lymph node, lung, bone
5	47	F	lymph node
