# Supplementary material for: Clinical and immunological evaluation of anti-apoptosis protein, survivin-derived peptide vaccine in phase I clinical study for patients with advanced or recurrent breast cancer
Source: J Transl Med. 2008 May 10;6:24. doi: 10.1186/1479-5876-6-24 (PMC2430193; doi:10.1186/1479-5876-6-24)
Supplement: Additional file 3 — Table 3: Outcome in the first protocol with survivin-2B peptide alone. This data showed the clinical and immunological evaluation in the first protocol. [file 1479-5876-6-24-S3.pdf]

Table 3 : Outcome in the first protocol with survivin-2B peptide alone

patient no.	dose of peptide (mg)	vaccination times	adverse event	Tumor marker (pre- / post-)	evaluation of CT image	DTH skin test	tetramer staining	ELISPOT assay
1	0.1	11	-	ICTP (7.2ng/ml / 5.5ng/ml)	PD †	+	undetected	-
2	0.1	4	-	CEA (6.1ng/ml / 14.9ng/ml)	PD	-	undetected	-
3	0.1	42	-	CA15-3 (323.7U/ml / 412.1U/ml)	SD ‡	-	undetected	-
4	0.1	5	-	CEA (10.3ng/ml / 28.6ng/ml)	PD	-	undetected	-
5	0.1	15	-	ICTP (7.8ng/ml / 10.5ng/ml)	SD	+	undetected	-
6	1.0	9	-	CA15-3 (53.4U/ml / 179.8 U/ml)	PD	-	undetected	-
7	1.0	3	-					
8	1.0	4	-	CEA (12.5ng/ml / 15.2ng/ml)	PD	-	detected	+
9	1.0	38	-	WNR ※		-	undetected	-
10	1.0	3	-					
11	1.0	4	-	CEA (22.5ng/ml / 45.2ng/ml)	PD	-	detected	-
12	1.0	5	-	CEA (47.4ng/ml / 81.3ng/nl)	PD	-	detected	-

tumor marker : pre- : before the 1st vaccination, post : after the 4th vaccination, WNR※ : within the normal range

evaluation of CT image : PD †: progressive disease, SD ‡: stable disease

DTH : + : positive reaction, - : negative reaction

tetramer staining : detected : an increase of twofold or more, undetected : a less than twofold increase

ELISPOT assay : + : many spots were visualized, - : spots were almost not visualized
