# Supplementary material for: Clinical and immunological evaluation of anti-apoptosis protein, survivin-derived peptide vaccine in phase I clinical study for patients with advanced or recurrent breast cancer
Source: J Transl Med. 2008 May 10;6:24. doi: 10.1186/1479-5876-6-24 (PMC2430193; doi:10.1186/1479-5876-6-24)
Supplement: Additional file 4 — Table 4: Outcome in the second protocol with survivin-2B peptide mixed IFA. This data showed the clinical and immunological evaluation in the second protocol. [file 1479-5876-6-24-S4.pdf]

Table 4 : Outcome in the second protocol with survivin-2B peptide mixed IFA

patient no.	dose of peptide (mg)	vaccination times	adverse event	Tumor marker (pre- / post-)	evaluation of CT image	DTH skin test	tetramer staining	ELISPOT assay
1	1.0	4	induration	CEA (4.9ng/ml / 7.4ng/ml)	PD	+	detected	-
2	1.0	3						
3	1.0	4	induration	CA15-3 (26.8U/ml / 79.7U/ml)	PD	-	detected	-
4	1.0	4	general malaise	CEA (23.2ng/ml / 33.9ng/ml)	PD	-	detected	-
5	1.0	4	general malaise fever	ICTP (11.8ng/ml / 28.0ng/ml)	PD	-	detected	+

tumor marker : pre- : before the 1st vaccination, post : after the 4th vaccination,

evaluation of CT image : PD : progressive disease,

DTH skin test : + : positive reaction, - : negative reaction

tetramer staining : detected : an increase of twofold or more, undetected : a less than twofold increase

ELISPOT assay : + : many spots were visualized, - : spots were almost not visualized
